# Optimal mean airway pressure during high-frequency oscillatory ventilation in an experimental model of acute respiratory distress syndrome: EIT-based method

**DOI:** 10.1186/s13613-020-0647-z

**Published:** 2020-03-06

**Authors:** Songqiao Liu, Zhanqi Zhao, Li Tan, Lihui Wang, Knut Möller, Inéz Frerichs, Tao Yu, Yingzi Huang, Chun Pan, Yi Yang, Haibo Qiu

**Affiliations:** 1 Department of Critical Care Medicine, Zhongda Hospital, School of Medicine, Southeast University, Jiangsu Province, Nanjing, 210009 China; 2 Institute of Technical Medicine, Furtwangen University, Jakob-Kienzle Strasse 17, 78054 VS-Schwenningen, Germany; 3 Department of Biomedical Engineering, Fourth Military Medical University, Xi’an, China; 4 Department of Critical Care Medicine, Beijing Tongren Hospital, Capital Medical University, Bejing, 100730 China; 5 Department of Anesthesiology and Intensive Care Medicine, University Medical Center of Schleswig–Holstein Campus Kiel, Arnold-Heller-Strasse 3, 24105 Kiel, Germany

**Keywords:** Acute respiratory distress syndrome, High-frequency oscillatory ventilation, Electrical impedance tomography, Mean airway pressure, Titration

## Abstract

**Background:**

High-frequency oscillatory ventilation (HFOV) may theoretically provide lung protective ventilation. The negative clinical results may be due to inadequate mean airway pressure (mPaw) settings in HFOV. Our objective was to evaluate the air distribution, ventilatory and hemodynamic effects of individual mPaw titration during HFOV in ARDS animal based on oxygenation and electrical impedance tomography (EIT).

**Methods:**

ARDS was introduced with repeated bronchoalveolar lavage followed by injurious mechanical ventilation in ten healthy male pigs (51.2 ± 1.9 kg). Settings of HFOV were 9 Hz (respiratory frequency), 33% (inspiratory time) and 70 cmH_2_O (∆pressure). After lung recruitment, the mPaw was reduced in steps of 3 cmH_2_O every 6 min. Hemodynamics and blood gases were obtained in each step. Regional ventilation distribution was determined with EIT.

**Results:**

PaO_2_/FiO_2_ decreased significantly during the mPaw decremental phase (*p* < 0.001). Lung overdistended regions decreased, while recruitable regions increased as mPaw decreased. The optimal mPaw with respect to PaO_2_/FiO_2_ was 21 (18.0–21.0) cmH_2_O, that is comparable to EIT-based center of ventilation (EIT-CoV) and EIT-collapse/over, 19.5 (15.0–21.0) and 19.5 (18.0–21.8), respectively (*p* = 0.07). EIT-CoV decreasing along with mPaw decrease revealed redistribution toward non-dependent regions. The individual mPaw titrated by EIT-based indices improved regional ventilation distribution with respect to overdistension and collapse (*p* = 0.035).

**Conclusion:**

Our data suggested personalized optimal mPaw titration by EIT-based indices improves regional ventilation distribution and lung homogeneity during high-frequency oscillatory ventilation.

## Background

Acute respiratory distress syndrome (ARDS) is common in ICU characterized by diffuse endothelial and epithelial injury, inflammatory pulmonary edema, small lung, lung injury inhomogeneities and severe hypoxemia [1, 2]. Mechanical ventilation remains mainstay in the management of patients with ARDS [3]. Lung protective ventilation with low tidal volumes [4], positive end-expiratory pressure (PEEP) [5, 6] and prone position [7] may improve outcomes. Nevertheless, the mortality of ARDS patients remains high, up to 30–50% [8].

High-frequency oscillatory ventilation (HFOV) delivered high mean airway pressure (mPaw) and extremely small tidal volumes to prevent alveolar derecruitment/overdistention as well as avoid the repeated opening/closing of individual alveolar [9]. Clinical trials [10] and large animal trials [11] have demonstrated that HFOV improves oxygenation, reduces lung inflammatory processes and histopathological damages, and attenuates oxidative lung injury compared with conventional mechanical ventilation (CMV).

Currently, clinical data do not support the use of HFOV in patients of ARDS. Two major multicenter, randomized trials (OSCAR and OSCILLATE) failed to show improvement on 30-day mortality in moderate-to-severe ARDS patients [12–14]. A meta-analysis found that HFOV might not improve outcome compared with CMV [15]. One possible reason may be the improper HFOV protocols applied and inadequate HFOV settings.

The optimal mPaw titration is still a challenge during HFOV. The selection of Paw is usually guided by a static *P*–*V* curve or based on the oxygenation index [9]; however, either computed tomography scanning [16] or frequent blood gas analysis is indispensable. Recently, a study showed that HFOV guided by transpulmonary pressure improved systemic hemodynamics, oxygenation, and lung overdistension compared with conventional HFOV in animals [17]. But the ventilation distribution and homogeneity remain unknown toward the methods mentioned above to titrate mPaw.

Electrical impedance tomography (EIT) might allow the clinician to better adjust these ventilatory settings. EIT is a bedside imaging technique that enables monitoring air distribution in the lungs [18]. Our previous study has showed the GI index may provide new insights into air distribution in CMV and may be used to guide ventilator settings [19, 20]. EIT might allow the clinician to better adjust ventilatory settings in HFOV. It is possible that HFOV would be safer and more effective with a more individualized approach to setting mPaw adjusted according to ventilation distribution bedside.

In the present study, our objective was to evaluate the air distribution, ventilatory, and hemodynamic effects of individual mPaw titration in HFOV based on oxygenation and EIT.

## Methods

The study was approved by the Science and Technological Committee and the Animal Use and Care Committee of the Southeast University, School of Medicine, Nanjing, China. All animal procedures and protocols were performed according to the Guidance for the Care and Use of Laboratory Animals [21].

### Animal preparation

A total of ten healthy male pigs (body weight 51.2 ± 1.9 kg, mean ± SD) were included. Pigs were anesthetized with an intramuscular injection of ketamine hydrochloride (3 mg/kg), atropine (2 mg/kg) and fentanyl citrate (2 mg/kg), followed by a continuous intravenous infusion of propofol (1–2 mg/kg/h), fentanyl citrate (0.5–1.0 μg/kg/h), midazolam (0.1 mg/kg/h), and atracurium (0.4 mg/kg/h). After the induction of anesthesia, the pigs were placed in supine position, on a thermo-controlled operation table to maintain body temperature at about 37.0 ℃. With local anesthesia, a mid-line neck incision was performed and the trachea was secured using an 8-mm-ID endotracheal tube. The animals received conventional mechanical ventilation (Servo-i ventilator, Solna, Sweden) under volume-controlled mode (respiratory rate 30 breaths per minute; inspiration-to-expiration time ratio 1:2 and PEEP 5 cmH_2_O; fraction of inspiration O_2_ (FiO_2_) and tidal volume (*V*_*T*_) 0.4 and 6 ml/kg, respectively). A Swan–Ganz catheter (Arrow International, Reading, PA, USA) was inserted through the internal jugular vein to measure central venous pressure (CVP) and pulmonary arterial wedge pressure (PAWP). A thermistor-tipped PiCCO catheter (Pulsion Medical System, Munich, Germany) was advanced through the right femoral artery to monitor the mean arterial pressure (MAP) and cardiac output (CO). In addition, arterial blood samples were collected from a PiCCO catheter. A continuous infusion of a 5 ml/(kg h) balanced electrolyte solution was administered during the experiment, and MAP was maintained above 60 mmHg with rapid infusions of 0.9% saline solution at up to 20 ml/kg, if required.

### Experimental protocol

After the initial animal preparation, the pigs were stabilized for 30 min and baseline measurements (*T*_Baseline_) were taken. ARDS was induced by repeated bilateral bronchoalveolar lavage with 30 ml/kg of isotonic saline (38 ℃). After stabilization, an arterial blood gas sample was obtained to verify that the ratio of partial pressure of arterial oxygen PaO_2_ and FiO_2_ decreased to less than 100 mmHg, followed by 1 h of injurious mechanical ventilation (PEEP 0 cmH_2_O and distending pressure 35 cmH_2_O in PCV). PaO_2_/FiO_2_ remained less than 100 mmHg for 30 min (*T*_ARDS_) with an increase of FiO_2_ to 1.0.

The mechanical ventilation mode was then switched to HFOV (FiO_2_ 1.0; respiratory frequency 9 Hz; inspiratory time 33%; ∆pressure 70 cmH_2_O), and a recruitment maneuver was performed (mPaw of 40 cmH_2_O for 40 s) after 15-min HFOV ventilation. After recruitment, stepwise mPaw decrements were performed from 36 to 9 cmH_2_O with a step of 3 cmH_2_O decrease every 6 min. (Flowchart of the study is showed in Additional file 1: Figure S1). CVP, PAWP, MAP and CO were recorded at every pressure level. All blood gas measurements were performed using an automated blood gas analyzer (Nova M; Nova Biomedical, Waltham, MA, USA).

### EIT measurements

Continuous EIT measurements started after tracheostomy (PulmoVista 500, Dräger Medical, Lübeck, Germany). An EIT electrode belt with 16 electrodes was placed around the thorax 5 cm above the xyphoid level and one reference ECG electrode was placed at the abdomen. The frequency of injected alternating current was selected automatically according to the noise spectrum. The images were continuously recorded and reconstructed at 40 Hz. The EIT data were reconstructed using a finite element method-based linearized Newton–Raphson reconstruction algorithm [22]. Baseline of the images was referred to the lowest impedance value measured during *T*_ARDS_. Oscillatory impedance variations of every 5 s were averaged to present the ventilation distribution. One-minute period at the end of each mPaw step was used for further EIT analysis.

### Mean paw titration strategies

#### mPaw optimization according to oxygenation

Optimal mPaw with respect to oxygenation was defined as mPaw in the step before the one at which PaO_2_ dropped by > 10% compared to previous step (Additional file 1: Figure S2).

#### mPaw optimization according to EIT-based center of ventilation (EIT-CoV)

The center of ventilation (CoV) index showing the vertical distribution of ventilation was calculated [23, 24]:

1 $$CoV = \frac{{\sum {(y_{i} \times I_{i} )} }}{{\sum {I_{i} } }} \times 100\%$$

*I*_*i*_ denotes impedance value of pixel *i*. *y*_*i*_ is the pixel height and pixel *i* is scaled so the most ventral row is 0 and the most dorsal row is 1. Optimal mPaw with respect to EIT-CoV was defined as mPaw associated with the CoV values closest to 50%. EIT-based COV index higher than 50% at high mPaw steps indicated ventilation distribution toward gravity-dependent regions.

#### mPaw optimization according to EIT-based collapse–overdistension

Recruitable regions compared to the highest mPaw level and overdistended regions were calculated using a method that was published recently [24]. During the analysis of HFOV in the present study, the oscillatory impedance variation was too small to confirm overdistension. Therefore, compared to the original method, the volume changes induced by mPaw changes were used. The differences of impedance between lower mPaw and higher mPaw were calculated. The regions with less than 20% changes were denoted as regions with limited volume changes. These regions with almost no pixels changes were considered to be overinflated, if they belonged to those image pixels that were showed in lung regions at lower mPaw step. Regions were considered to be recruitable if they were included in the lung regions at end-expiration at the highest mPaw step but not at the current mPaw step.

The lung regions at mPaw level *n* were defined as pixels with higher impedance value (*I*) than 20% of maximum changes compared to the lowest mPaw level *r* (reference level, the lowest mPaw level).

2 $$j \in {\text{Lung if }}I_{j} > 20\% \times \hbox{max} \left( {I_{n\;,\;i} - I_{r\;,\;i} } \right), i \in \left[ {1, 1024} \right]$$

Subsequently, the maximum differences of impedance (*I*_max-diff_) between lower mPaw (denoted as mPaw level *n*) and higher mPaw (mPaw level *n* + 1) were calculated.

3 $$I_{{{ \hbox{max} }\; - \;{\text{diff}}}} = \hbox{max} \left( {I_{n\; + \;1\;,\;i} - I_{n\;,\;i} } \right), i \in \left[ {1, 1024} \right]$$

The regions with less than 20% changes were denoted as regions with limited volume changes (for pixel *k*, *k *∈ *i*, *I*_*k*_ < 20% × *I*_max-diff_). These regions *k* were compared to lung regions at mPaw level n (*j*_*n*_). They were considered to be overinflated, if they belonged to lung regions at mPaw step *n* at the same time (*k *∩ *j*_*n*_ intersection of set *k* and set *j*_*n*_).

The numbers of pixels in these two regions were plotted against decremental mPaw. Optimal mPaw with respect to recruitable and overdistended regions was defined as the step where these two-pixel curves intersected. If the curves not intersected, mPaw with the lowest sum of recruitable and overdistended regions was selected. With the nature of this method, no values could be calculated for the lowest mPaw step, since the calculation required a comparison with a lower mPaw step (Eq. 3). Overdistension/recruitment ratio was defined as number of pixels in the overdistended regions over that in the recruitable regions.

### Statistical analysis

Statistical analysis was performed with the MATLAB software package (MATLAB 7.2 statistic toolbox, The MathWorks Inc., Natick, MA, USA). Due to the limited number of subjects, results are presented as median ± interquartile range. One-way Kruskal–Wallis test was used to assess the significance of differences in Hemodynamics and oxygenation among different mPaw, and differences in optimal mPaw estimated with various criteria. A *p* value lower than 0.05 was considered statistically significant. Wilcoxon signed-rank test was applied for further comparison within groups and the significance levels were corrected for multiple comparisons using Holm’s sequential Bonferroni method.

## Results

ARDS was successfully induced by repeated bronchoalveolar lavages in all 10 pigs. The induction of ARDS led to a significant decrease in PaO_2_/FiO_2_ (*p* < 0.001).

### Hemodynamics

MAP and CO increased while CVP and PAWP decreased along with the decremental mPaw trial. Hemodynamic data during the mPaw trial are plotted in Additional file 1: Table S1.

### Titration of optimal mPaw by oxygenation

The effect of mPaw on the PaO_2_/FiO_2_ and partial pressure of arterial carbon dioxide (PaCO_2_) during HFOV are shown in Additional file 1: Figure S2. During the decremental phase, significant decrease in PaO_2_/FiO_2_ and increase in PaCO_2_ were found between the mPaw step of 18 cmH_2_O and 15 cmH_2_O (*p* < 0.001) (Additional file 1: Figure S2 left). The optimal mPaw calculated by individual animal with respect to PaO_2_/FiO_2_ was 21 (18.0–21.0) cmH_2_O.

### Optimal mPaw derived from regional ventilation distribution

**Fig. 1:**
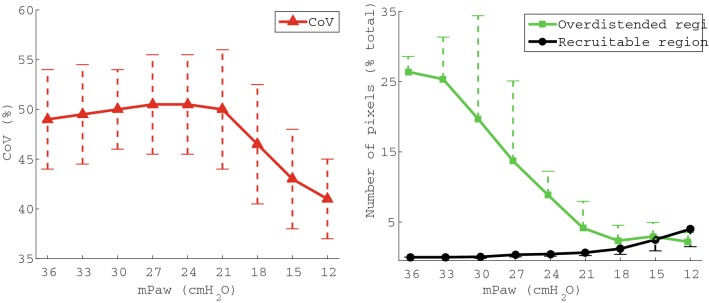
The change of EIT-based measures center of ventilation (CoV, left) and overdistended and recruitable regions (right) along with decremental mPaw titration in HFOV

CoV decreased along with mPaw decrease revealing a redistribution of ventilation toward non-dependent regions (Fig. 1, left). The optimal mPaw with respect to EIT-CoV in all pigs was 19.5 (15.0–21.0) cmH_2_O and the values among individuals varied a lot.

EIT-derived overdistended regions decreased as mPaw decreased (Fig. 1, right, green circles). At the same time, recruitable regions increased (black stars). The optimal mPaw using the approach based on the calculated EIT-collapse/over was 19.5 (18.0–21.8) cmH_2_O.

### Optimal mPaw derived from different methods

**Fig. 2:**
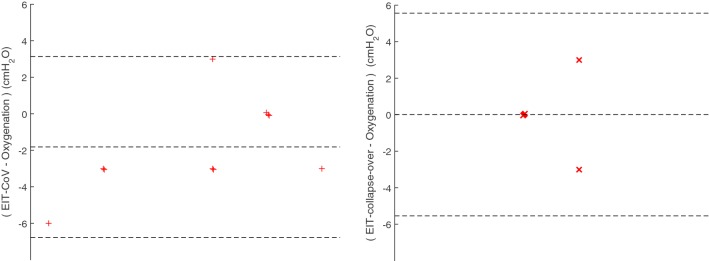
Bland–Altman plots comparing optimal mPaw settings with EIT-CoV and oxygenation (left), with EIT-collapse-overdistended and oxygenation (right). EIT-CoV: EIT-based center of ventilation; EIT-collapse-overdistended: EIT-based overdistended and recruitable regions

**Fig. 3:**
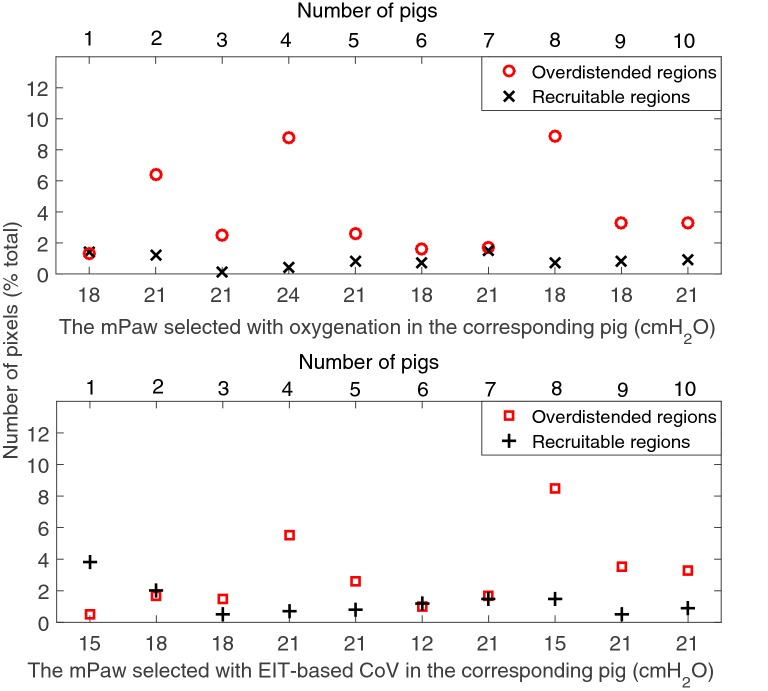
The recruitable regions and overdistended regions in mPaw selected with oxygenation (left) and EIT-based CoV (right) in the individual pig. Number of pixels is presented as Black asterisk (recruitable lung region) and red circles (overdistended lung region) with the optimal mPaw were defined with oxygenation (Upper). The number of pixels is presented as black crosses (recruitable lung region) and red squares (overdistended lung region) with the optimal mPaw were defined with EIT-based center of ventilation index (lower *x*-axis) as well (lower)

The optimal mPaw with respect to PaO_2_/FiO_2_ was 21 (18.0–21.0) cmH_2_O, that is comparable to EIT-based center of ventilation (EIT-CoV) and EIT-collapse/over, 19.5 (15.0–21.0) and 19.5 (18.0–21.8), respectively (*p* = 0.07). The differences between the selected mPaw according to oxygenation and according to “EIT-Cov” and “EIT-collapse/over” were compared with Bland–Altman plots (Fig. 2). The differences in mPaw selection between oxygenation and EIT-based methods could be as high as 6 cmH_2_O in some pigs. The optimal mPaw settings derived from oxygenation, EIT-CoV and EIT-collapse/over were compared (Table 1). In Fig. 3, overdistended and recruitable regions at mPaw levels selected based on oxygenation were illustrated. In each pig, the optimal mPaw defined with oxygenation was given (*x*-axis). The mPaw titrated by EIT-based indices improved regional air distribution with respect to overdistension and collapse (comparison among 3 mPaw titration strategies, *p* = 0.035) (Table 2).

**Table 1:** Selected mPaw according to oxygenation, EIT-CoV and EIT-regions

Pig number	mPaw (cmH_2_O) selected by		
	Oxygenation	EIT-CoV	EIT-regions
1	18	15	18
2	21	18	18
3	21	18	21
4	24	21	18
5	21	21	24
6	18	12	18
7	21	21	21
8	18	15	18
9	18	21	21
10	21	21	24
Median (interquartile range)	21 (18.0–21.0)	19.5 (15.0–21.0)	19.5 (18.0–21.8)
F	3.115		
*P*	0.07

**Table 2:** The effects of the 3 mPaw titration strategies on oxygenation, overdistension/recruitment and hemodynamics

mPaw titration strategies	Oxygenation	EIT-CoV	EIT-regions	*F*	*p*
PaO_2_/FiO_2_ (mmHg)	210.8 ± 38.5	176.1 ± 47.7	204.7 ± 43.1	1.833	0.179
Overdistension/recruitment ratio	8.1 ± 7.9	5.3 ± 4.7	2.8 ± 2.1	4.985	0.035*
HR (BPM)	84.6 ± 30.5	82.2 ± 27.8	85.5 ± 28.9	0.0345	0.966
MAP (mmHg)	90.3 ± 33.8	92.5 ± 35.5	91.7 ± 34.1	0.0104	0.990
CVP (mmHg)	8.9 ± 2.8	8.1 ± 2.3	8.2 ± 3.0	1.879	0.183
PAWP (mmHg)	11.4 ± 2.9	10.9 ± 2.8	11.1 ± 3.1	0.774	0.457
CO (L/min)	4.6 ± 1.4	4.6 ± 1.5	4.5 ± 1.2	0.0155	0.985

Mean values and standard deviations are shown

*HR* heart rate, *BPM* breaths per minute, *MAP* mean arterial pressure, *CVP* measure central venous pressure, *CO* Cardiac output, *PAWP* pulmonary arterial wedge pressure

*p < 0.05

## Discussion

In the present study, novel EIT-based method titrating mPaw under HFOV was proposed and evaluated in ARDS model. The titration results were compared with oxygenation method and the effects on lung homogeneity were examined. We found that the individual mPaw titrated by EIT-based indices improved regional ventilation distribution with respect to overdistension and collapse and the suggested mPaw may not always match the ones proposed by oxygenation method.

HFOV may remain a tool in managing patients with severe ARDS and refractory hypoxemia and not the first-line treatment for ARDS patient. HFOV with high mPaw values applied in both two trials [25, 26] might contribute to negative clinical outcome on ARDS patients and canceled out the positive effects. HFOV using Paw set according to a static *P*–*V* curve [16], oxygenation, mean airway pressure during CMV [27], and transpulmonary pressure [17] has been examined in clinical and animal studies, but the bedside monitoring base on ventilation distribution is lacking. In the present study, we provide new mPaw titration method in respect of regional ventilation distribution that improves lung homogeneity. The increased mPaw lead to more lung tissue hyperinflated, and the EIT-CoV decrease, which revealed redistribution toward non-dependent regions. A critical issue of this EIT-based method was the pre-defined threshold used to identify lung regions. Further studies are required to confirm if the threshold used in the present study is optimal for various subjects and conditions.

The reliability of EIT has been confirmed and EIT has been used in clinic setting and adjust of CMV. EIT has been used in PEEP titration and tidal volume setting by comparison with various conventional methods, such as CT [28], single-photon-emission computed tomography [29], positron emission tomography [30], and pneumotachography [31]. Previous studies have already shown that EIT was able to monitor ventilation distribution during HFOV in preterm infants and patients with chronic obstructive pulmonary disease [22, 32]. The optimal settings based on oxygenation were comparable to EIT-CoV and EIT-regional ventilation distribution. It was also observed that overdistended regions were large at the mPaw selected with oxygenation method in several pigs. PF ratio is an invasive method with a certain time delay in response to pressure changes. Although the average values between EIT-derived measures were not very different, individual differences could be large (up to 6 cmH_2_O, Figs. 2 and 3). Hence, mPaw titration with EIT-based indices improved regional ventilation distribution while titration aiming oxygenation was not always the case. Besides, it is worth to note that EIT is currently the only bedside non-invasive tool to assess overdistension. Further investigation should be conducted in future clinical studies.

Our study has some limitations. First, as an experimental study, these data were obtained in animals and its clinical impact may be limited. Therefore, the optimal mPaw selected in the present study might be not suitable with that in ARDS patients. Second, HFOV should not be employed in the absence of well-trained expertise because of its complexity. Further validation study to assess the feasibility of such strategies in ARDS patients with proposed method should be conducted.

## Conclusion

Our data provide personalized optimal mPaw titration in HFOV with EIT-based indices, which may provide a new insight of regional ventilation distribution and lung homogeneity during high-frequency oscillatory ventilation.

## Supplementary information

**Additional file 1: Figure S1.** Flowchart of the study. **Figure S2.** PaO_2_/FiO_2_ (left) and PaCO_2_ (right) during mPaw decrements trial after having fully recruited the lungs.

**Additional file 2: Table S1.** Hemodynamics characteristics during decremental HFOV mPaw (n = 10).

## Data Availability

The datasets used and/or analyzed during the current study are available from the corresponding author on reasonable request.

## Abbreviations

ARDS: Acute respiratory distress syndrome
CO: Cardiac output
CoV: Center of ventilation
CT: Computed tomography
CVP: Central venous pressure
EELI: End-expiratory lung impedance
EIT: Electrical impedance tomography
FiO_2_: Fraction of inspired oxygen
HFOV: High-frequency oscillatory ventilation
MAP: Mean arterial pressure
mPaw: Mean airway pressure
PaCO_2_: Partial pressure of CO_2_ in arterial blood
PaO_2_: Partial pressure of O_2_ in arterial blood
PAWP: Pulmonary arterial wedge pressure
PEEP: Positive end-expiratory pressure
VILI: Ventilator-induced lung injury
VT: Tidal volume

## References

[1] Ashbaugh DG, Bigelow DB, Petty TL, Levine BE (1967). Acute respiratory distress in adults. Lancet.

[2] Force ADT, Ranieri VM, Rubenfeld GD, Thompson BT, Ferguson ND, Caldwell E (2012). Acute respiratory distress syndrome: the Berlin definition. JAMA.

[3] Thompson BT, Chambers RC, Liu KD (2017). Acute respiratory distress syndrome. N Engl J Med.

[4] Brower RG, Matthay MA, Morris A, Schoenfeld D, Thompson BT, Acute Respiratory Distress Syndrome N (2000). Ventilation with lower tidal volumes as compared with traditional tidal volumes for acute lung injury and the acute respiratory distress syndrome. N Engl J Med..

[5] Guo L, Xie J, Huang Y, Pan C, Yang Y, Qiu H (2018). Higher PEEP improves outcomes in ARDS patients with clinically objective positive oxygenation response to PEEP: a systematic review and meta-analysis. BMC Anesthesiol..

[6] Chiumello D, Cressoni M, Carlesso E, Caspani ML, Marino A, Gallazzi E (2014). Bedside selection of positive end-expiratory pressure in mild, moderate, and severe acute respiratory distress syndrome. Crit Care Med.

[7] Guerin C, Reignier J, Richard JC, Beuret P, Gacouin A, Boulain T (2013). Prone positioning in severe acute respiratory distress syndrome. N Engl J Med.

[8] Bellani G, Laffey JG, Pham T, Fan E, Brochard L, Esteban A (2016). Epidemiology, patterns of care, and mortality for patients with acute respiratory distress syndrome in intensive care units in 50 countries. JAMA.

[9] Chan KP, Stewart TE, Mehta S (2007). High-frequency oscillatory ventilation for adult patients with ARDS. Chest.

[10] Sud S, Sud M, Friedrich JO, Meade MO, Ferguson ND, Wunsch H (2010). High frequency oscillation in patients with acute lung injury and acute respiratory distress syndrome (ARDS): systematic review and meta-analysis. BMJ.

[11] Goffi A, Ferguson ND (2014). High-frequency oscillatory ventilation for early acute respiratory distress syndrome in adults. Curr Opin Crit Care.

[12] Ferguson ND, Cook DJ, Guyatt GH, Mehta S, Hand L, Austin P (2013). High-frequency oscillation in early acute respiratory distress syndrome. N Engl J Med.

[13] Malhotra A, Drazen JM (2013). High-frequency oscillatory ventilation on shaky ground. N Engl J Med.

[14] Young D, Lamb SE, Shah S, MacKenzie I, Tunnicliffe W, Lall R (2013). High-frequency oscillation for acute respiratory distress syndrome. N Engl J Med..

[15] Goligher EC, Munshi L, Adhikari NKJ, Meade MO, Hodgson CL, Wunsch H (2017). High-frequency oscillation for adult patients with acute respiratory distress syndrome. A systematic review and meta-analysis. Ann Am Thorac Soc..

[16] Luecke T, Meinhardt JP, Herrmann P, Weisser G, Pelosi P, Quintel M (2003). Setting mean airway pressure during high-frequency oscillatory ventilation according to the static pressure–volume curve in surfactant-deficient lung injury: a computed tomography study. Anesthesiology.

[17] Klapsing P, Moerer O, Wende C, Herrmann P, Quintel M, Bleckmann A (2018). High-frequency oscillatory ventilation guided by transpulmonary pressure in acute respiratory syndrome: an experimental study in pigs. Crit Care.

[18] Frerichs I, Amato MB, van Kaam AH, Tingay DG, Zhao Z, Grychtol B (2017). Chest electrical impedance tomography examination, data analysis, terminology, clinical use and recommendations: consensus statement of the TRanslational EIT developmeNt stuDy group. Thorax.

[19] Zhao Z, Moller K, Steinmann D, Frerichs I, Guttmann J (2009). Evaluation of an electrical impedance tomography-based Global Inhomogeneity Index for pulmonary ventilation distribution. Intensive Care Med.

[20] Zhao Z, Steinmann D, Frerichs I, Guttmann J, Moller K (2010). PEEP titration guided by ventilation homogeneity: a feasibility study using electrical impedance tomography. Crit Care.

[21] Guide for the Care and Use of laboratory animals. Washington (DC):National Academies Press (US). 2011. 21595115

[22] Gong B, Krueger-Ziolek S, Moeller K, Schullcke B, Zhao Z (2015). Electrical impedance tomography: functional lung imaging on its way to clinical practice?. Expert Rev Respir Med..

[23] Frerichs I, Hahn G, Golisch W, Kurpitz M, Burchardi H, Hellige G (1998). Monitoring perioperative changes in distribution of pulmonary ventilation by functional electrical impedance tomography. Acta Anaesthesiol Scand.

[24] Liu S, Tan L, Moller K, Frerichs I, Yu T, Liu L (2016). Identification of regional overdistension, recruitment and cyclic alveolar collapse with electrical impedance tomography in an experimental ARDS model. Crit Care.

[25] Sklar MC, Fan E, Goligher EC (2017). High-frequency oscillatory ventilation in adults with ARDS: past, present, and future. Chest.

[26] Ng J, Ferguson ND (2017). High-frequency oscillatory ventilation: still a role?. Curr Opin Crit Care..

[27] Guervilly C, Forel JM, Hraiech S, Demory D, Allardet-Servent J, Adda M (2012). Right ventricular function during high-frequency oscillatory ventilation in adults with acute respiratory distress syndrome. Crit Care Med.

[28] Wrigge H, Zinserling J, Muders T, Varelmann D, Gunther U, von der Groeben C (2008). Electrical impedance tomography compared with thoracic computed tomography during a slow inflation maneuver in experimental models of lung injury. Crit Care Med.

[29] Frerichs I, Hinz J, Herrmann P, Weisser G, Hahn G, Dudykevych T (2002). Detection of local lung air content by electrical impedance tomography compared with electron beam CT (Bethesda, Md: 1985). J Appl Physiol..

[30] Richard JC, Pouzot C, Gros A, Tourevieille C, Lebars D, Lavenne F (2009). Electrical impedance tomography compared to positron emission tomography for the measurement of regional lung ventilation: an experimental study. Crit Care.

[31] Victorino JA, Borges JB, Okamoto VN, Matos GF, Tucci MR, Caramez MP (2004). Imbalances in regional lung ventilation: a validation study on electrical impedance tomography. Am J Respir Crit Care Med.

[32] Miedema M, de Jongh FH, Frerichs I, van Veenendaal MB, van Kaam AH (2011). Changes in lung volume and ventilation during surfactant treatment in ventilated preterm infants. Am J Respir Crit Care Med.

