# Supplementary material for: Optimal mean airway pressure during high-frequency oscillatory ventilation in an experimental model of acute respiratory distress syndrome: EIT-based method
Source: Ann Intensive Care. 2020 Mar 6;10:31. doi: 10.1186/s13613-020-0647-z (PMC7060304; doi:10.1186/s13613-020-0647-z)
Supplement: Supplementary file 1 — Additional file 1: Figure S1. Flowchart of the study. Figure S2. PaO2/FiO2 (left) and PaCO2 (right) during mPaw decrements trial after having fully recruited the lungs. [file 13613_2020_647_MOESM1_ESM.pdf]

Optimal mean airway pressure during high frequency oscillatory ventilation in an experimental model of acute respiratory distress syndrome：EIT-based method

Songqiao Liu, MD, PhD [1], Zhanqi Zhao, PhD [2, 3], Li Tan, MD, [1, 4], Lihui Wang, MD [1], Knut Möller, MD, PhD [2], Inez Frerichs, MD, PhD [5], Tao Yu, MD, PhD [1], Yingzi Huang, MD, PhD [1], Chun Pan MD, PhD[1], Yi Yang, MD, PhD [1], and Haibo Qiu, MD, PhD [1§]

Additional File 1

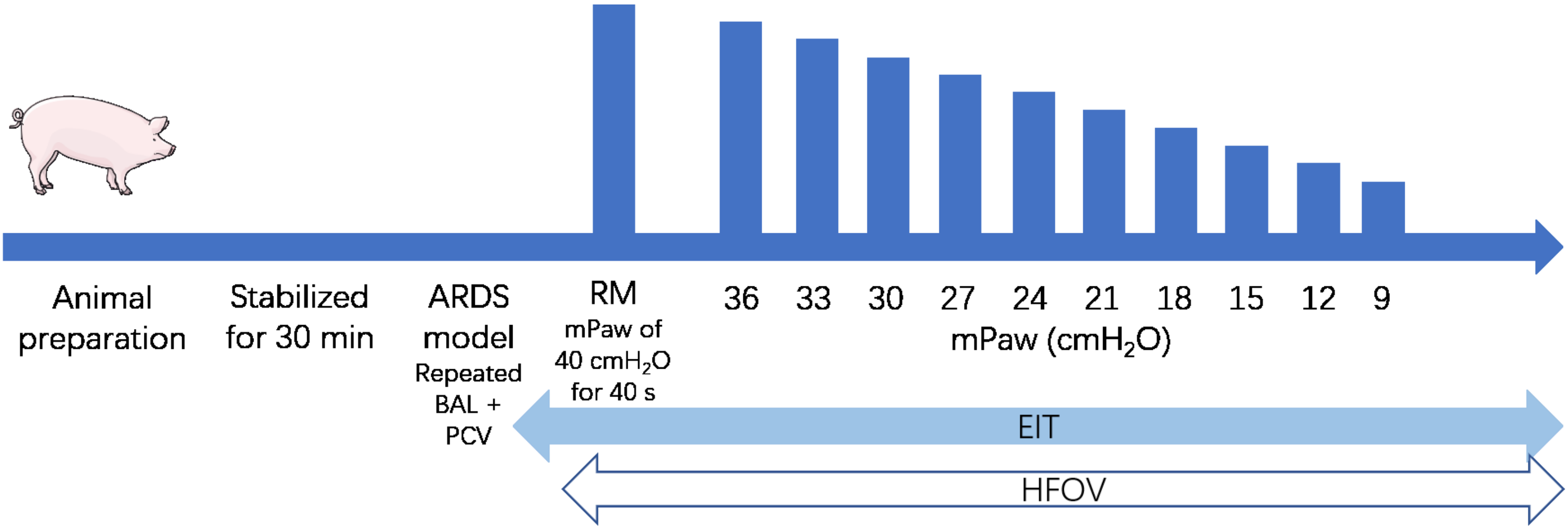

Additional file 1: Figure S1. Flowchart of the study.

ARDS, acute respiratory distress syndrome; EIT, electrical impedance tomography; HFOV, high-frequency oscillatory ventilation; mPaw, mean airway pressure; PCV, pressure control ventilation.

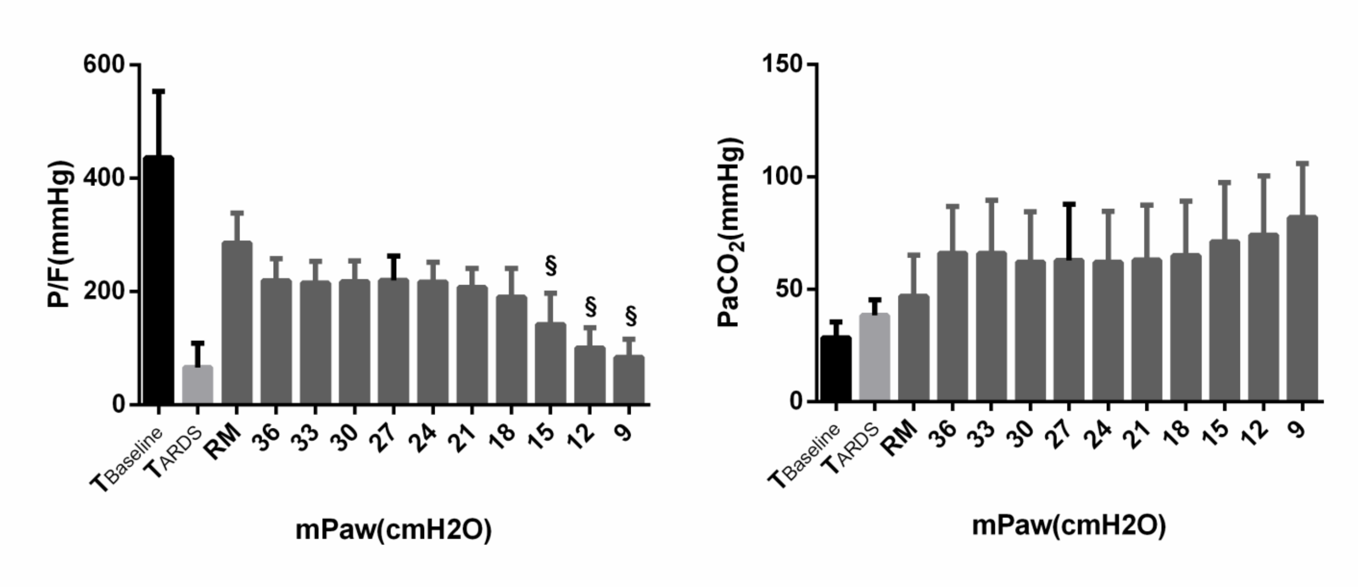

Additional file 1: Figure S2. PaO_2/FiO_2 (left) and $PaCO_2$ (right) during mPaw decrements trial after having fully recruited the lungs.

Mean values and standard deviations are shown.

§, PaO_2/FiO_2 decrease >10% compared with mPaw 18 cmH_2O, indicates the mPaw level at which the predefined optimal oxygenation criteria were reached during mPaw decrements trial after having fully recruited the lungs.
