# Supplementary material for: Optimal mean airway pressure during high-frequency oscillatory ventilation in an experimental model of acute respiratory distress syndrome: EIT-based method
Source: Ann Intensive Care. 2020 Mar 6;10:31. doi: 10.1186/s13613-020-0647-z (PMC7060304; doi:10.1186/s13613-020-0647-z)
Supplement: Supplementary file 2 — Additional file 2: Table S1. Hemodynamics characteristics during decremental HFOV mPaw (n = 10). [file 13613_2020_647_MOESM2_ESM.docx]

**Optimal mean airway pressure during high frequency oscillatory ventilation in an experimental model of acute respiratory distress syndrome：EIT-based method**

Songqiao Liu, MD, PhD ^1^, Zhanqi Zhao, PhD ^2, 3^, Li Tan, MD, ^1, 4^, Lihui Wang, MD ^1^, Knut Möller, MD, PhD ^2^, Inez Frerichs, MD, PhD ^5^, Tao Yu, MD, PhD ^1^, Yingzi Huang, MD, PhD ^1^, Chun Pan MD, PhD^1^, Yi Yang, MD, PhD ^1^, and Haibo Qiu, MD, PhD ^1§^

Additional File 2

Table S1 Hemodynamics characteristics during decremental HFOV mPaw（n=10）

|  | T_Baseline_ | T_ARDS_ | mPaw(cmH_2_O) | | | | | | | | | |
| --- | --- | --- | --- | --- | --- | --- | --- | --- | --- | --- | --- | --- |
|  |  |  | 36 | 33 | 30 | 27 | 24 | 21 | 18 | 15 | 12 | 9 |
| HR(BPM) | 67(60 -73) | 80 (73 -86) | 101(85-111) ^*^ | 103(82-118)^*^ | 96(83-121)^*^ | 88(73-103)^*^ | 80(73 -101) | 76(66 -102) | 70(65 -104) | 69(57 -108) | 70(59 -110) | 85(61 -114) |
| MAP(mmHg) | 116(108-124) | 127(124-133) | 66(65-74)^*^ | 69(64-78)^*^ | 75(67-98)^*^ | 84(79-108)^*^ | 96(81-110)^*^ | 103(90-109)^*^ | 104(94-111)^*^ | 104(92-109)^*^ | 108(102-113) | 106(103-113) |
| CVP(mmHg) | 4.5(3.3-6.8) | 8.0(6.0-8.0) | 9.5(6.8-10.8)^*^ | 10.0(7.0-11.0)^*^ | 9.5(8.0-10.1)^*^ | 9.5(8.0-10.1)^*^ | 9.5(8.0-10.1)^*^ | 8.5(7.3-10.0)^*^ | 8.0(6.0-9.0)^*^ | 7.5(5.3-9.0) | 7.0(5.0-8.0) | 6.0(3.8-8.3) |
| PAWP(mmHg) | 7.0(4.3-8.7) | 10.0(8.0-11.0) | 13.0(9.0-19.5)^*^ | 15.0(1.0-17.0)^*^ | 14.5(12.2-15.7)^*^ | 13.5(12.0-15.0)^*^ | 13.5(11.2-14.0)^*^ | 12.5(10.3-14.0)^*^ | 11.5(8.7 -12.8)^*^ | 10.0(7.5-12.0) | 9.0(7.0-11.0) | 7.0(4.5-8.5) |
| CO(L/min) | 5.4(5.0-5.7) | 4.7(4.6-4.8) | 3.1(2.5-4.1)^*^ | 3.5(3.2-4.2) ^*^ | 3.8(3.5-4.0)^*^ | 4.1(3.5-5.0) | 4.3(4.1-5.1) | 4.7(4.0-5.4) | 5.0(4.1-5.8) | 5.2(4.3-6.0) | 5.3(4.6-5.9) | 5.5(5.2-5.8) |

Median (Interquartile range) are shown. *p< 0.05, Compared with baseline measurements.

HR, heart rate; BPM, breaths per minute; MAP, mean arterial pressure; CVP, measure central venous pressure; CO, Cardiac output; PAWP, pulmonary arterial wedge pressure. T_Baseline_, during baseline measurements; T_ARDS_, after induction of ARDS.
